# Immediate Effects of Tibialis Anterior and Calf Muscle Taping on Center of Pressure Excursion in Chronic Stroke Patients: A Cross-Over Study

**DOI:** 10.3390/ijerph17114109

**Published:** 2020-06-09

**Authors:** Shin Jun Park, Tae-Hyun Kim, Seunghue Oh

**Affiliations:** 1 Department of Physical Therapy, Gangdong University, 278, Daehak-gil, Gamgok-myeon, Eumseong-gun, Chungcheongbuk-do 27600, Korea; 3178310@naver.com; 2 NEULBOM Hospital 17, Poeun-daero 59 beon-gil, Suji-gu, Yongin-si, Gyeonggi-do 16864, Korea; tohyunna2@naver.com; 3 Department of Physical Therapy, Graduate School, Dankook University, 119, Dandae-ro, Dongnam-gu, Cheonan-si, Chungnam 330-714, Korea

**Keywords:** kinesiology tape, stroke, ankle control, center of pressure

## Abstract

Stroke patients often have muscles spasticity, difficulty with posture control, and tend to fall. This study investigated the use of kinesiology tape for patients with spasticity of ankle muscles after stroke. This study had a randomized, repeated measures design, and evaluated the immediate effect of kinesiology tape on the center of pressure (COP) excursion when applied to the calf and tibialis anterior muscles in stroke survivors. We determined that the taping attachment direction affects the COP movement. Twenty subjects were randomly assigned to the tibialis anterior taping condition, calf taping condition, or nontaping condition. Condition excursion was assessed. The measured variables included the paretic side area, nonparetic side area, forward area, and backward area of COP. All evaluations were conducted immediately after taping. COP excursion for chronic stroke survivors improved after tibialis anterior and calf taping (*p* < 0.05). Calf taping conditions increased significantly in the forward area (*p* < 0.05), and tibialis anterior taping conditions increased significantly in the backward area (*p* < 0.05). Kinesiology tape immediately increased the forward and backward COP excursion for patients with stroke.

## 1. Introduction

Stroke patients with neurological impairment experience 43% more muscle spasticity [1] and 56% more somatosensory deficits in the affected legs [2]. Stroke patients with poor postural control experience approximately 40% more falls per year [3]. Patients who are hemiparalyzed after stroke experience abnormal muscle tone changes due to neurological damage, decreased muscle strength, and an inaccurate range of motion in the joints. In addition, they have difficulty with posture control due to a lack of sensory processing abilities [4].

Spastic hypertonia at the ankle joint is a typical problem for after-stroke patients [5]. Ankle spasticity affects the functional performance regarding passive biomechanic properties and internal ankle joint torque during functional movement, such as posture control for balance and gait [5,6].

Motion strategies for maintaining posture control include ankle and buttock strategies. Ankle strategies involving upper and lower trunk and hip joint are focused on moving in the same direction while standing straight [7]. Spasticity of the ankle muscle, such as the tibialis anterior and calf muscles in stroke patients, is more often compensated for through hip strategies instead of ankle strategies [4]. Compared to healthy individuals, stroke patients use compensatory strategies more often, such as holding onto objects or a wall, or hip strategies to move and maintain balance [4]. In fact, because the supporting surface is decreased when the ankle muscle is spastic, patients are unable to use ankle strategies [8,9].

Various intervention methods are used so that patients lacking balancing abilities can use ankle strategies and improve posture control. Taping is one such method [10]. There are various taping methods, such as rigid taping, elastic taping (kinesiology taping), and spiral taping. Among them, the kinesiology taping method is used to enhance, support, and move the residual muscles [11]. Previous studies report that taping promoted the skin receptors, promoted ankle functions by supporting the movements of stroke patients, and resulted in improved balance ability [12,13]. Moreover, depending on how the tape is attached, taping increases movement (i.e., mobility) [14]. 

Although ankle joint movement is crucial for functional movement and kinesiology tape is useful, sufficient research has not been performed to confirm the characteristics of elastic taping. Therefore, we intend to identify the center of pressure (COP) change according to the taping attachment method by attaching taping to the tibialis anterior [12] and gastrocnemius [15,16].

## 2. Materials and Methods

### 2.1. Participants

This study recruited participants diagnosed with a stroke at Rehabilitation Hospital in Gyeonggi-do. A cross-over study design was approved by the Institutional Review Board (Institutional Research Review Committee: 2-1040966-AB-N-01-20-1810-HSR-114-9). The inclusion criteria were as follows: (1) first-ever stroke; (2) six months or longer since the stroke diagnosis; (3) Brunnstrom’s stage 3–5 for motor recovery in the affected lower limb; (4) modified Ashworth scale score ≤2 for the gastrocnemius; (5) passive dorsiflexion range of motion in the supine position of 0 or higher; (6) ability to stand straight for at least one minute without assistance; (7) no orthopedic disease in the ankle joint at any time; (8) no disabilities in vision, hearing, or vestibular senses; (9) score of ≥24 points for the Mini-Mental State Examination; and (10) normal score for the pin-prick test. The exclusion criteria were as follows: (1) neurological issues other than stroke that may interfere with balance control, (2) pain that may impact daily life, and (3) skin rashes or allergies associated with the tape. All participants were given the full instructions of the study and agreed to participate in the experiment.

### 2.2. Sample Size Calculation

The sample size of this study was determined using G-power software (G* Power 3.1.9.2, Heinrich–Heine–Universität, Düsseldorf, Germany). The pilot studies were conducted with six participants. According to six pilot studies, the partial η^2^ was 0.333, which became 0.71 when the effect size was calculated. Therefore, for a total sample size of 5, the effect size was 0.71, error probe was 0.05, power was 0.8, number of groups was 1, number of measurements was 3, correlation among representative measures was 0.5, and nonsphericity correction ε was 1. 

### 2.3. Study Design

This study used a randomized, repeated measures design (a cross-over study design). The center of pressure (COP) of all participants was measured in random order. Participants were randomly assigned to the tibialis anterior tape condition, calf tape condition, or nontape condition. There was no order for the tape application; it was attached and evaluated at random. These participants randomly picked a paper marked A, B, or C to determine the condition in which they would be included. A was the tibialis anterior taping condition, B was the calf taping condition, and C was the nontaping condition. The double-blind method was used to ensure objectivity and exclude researcher and participant bias. No information about the taping effects was given to the study participants. The researcher was blinded to the outcomes of the evaluation. Three physical therapists, blinded to the study, were trained to tape all participants in the same way.

### 2.4. Taping Method

The tape used in this study was an adhesive kinesiology tape (Kinesiology 3NS Tape; TS, Korea) and was cut to match the length required for each participant. The length was cut by 25% shorter than the required length. Note that kinesiology taping requires stretching when applied; a taping 1/4 stretch or 1/8 stretch is usually used. In our study, tape with elasticity of 25% (1/4 stretch) was used when the tape was stretched and attached to the full-length tape. On taping, no stretch tension was applied to the origin and insertion of each tape to avoid a certain amount of pull on the skin [17]. Stretch tension was applied to only the middle part of the tape.

### 2.5. Tibial Muscle Taping

**Figure 1:**
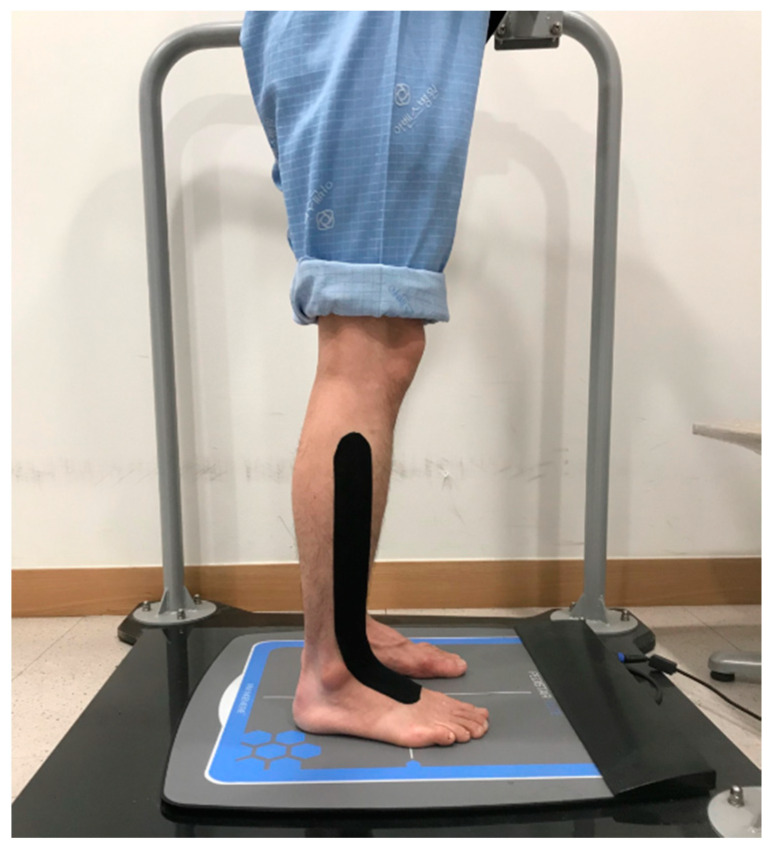
Tibial muscle taping.

Figure 1 shows how the tape was attached to the tibial muscle. While the participant was lying down, the tape was attached from the outside of the surface of the tibia, which connects the tibial muscle, to the first metatarsal bone [12]. 

### 2.6. Calf Muscle Taping

**Figure 2:**
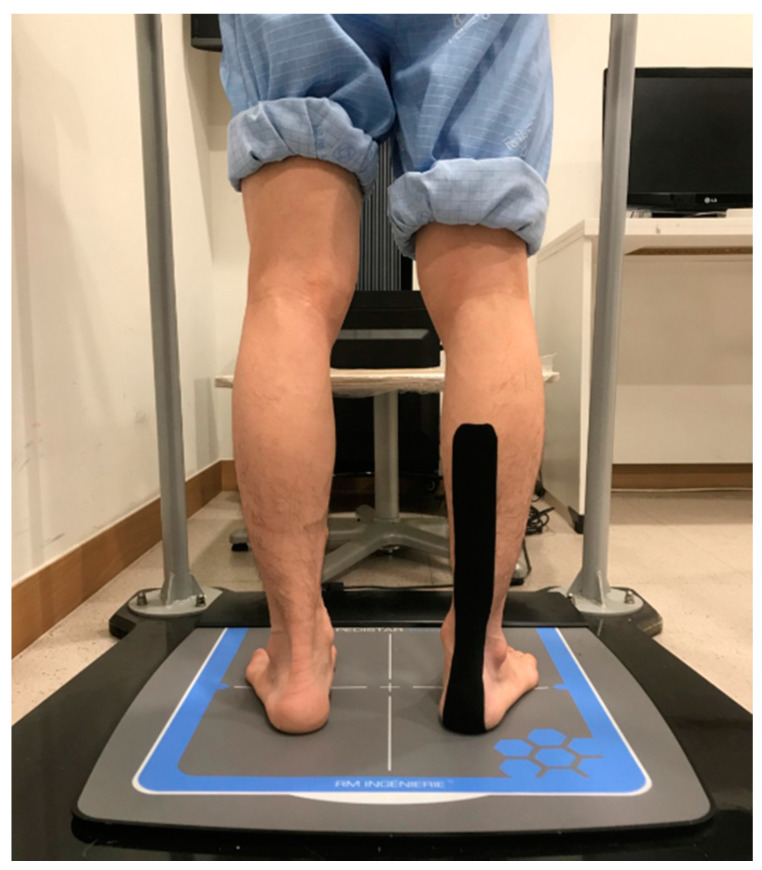
Calf muscle taping.

Figure 2 shows how the tape was attached to the calf muscle. While participants were lying face-down, they bent the knee joint 90 degrees. The tape was attached from the bottom of the foot at the heel to one-third of the calf [10].

### 2.7. Measurement Procedures

Each participant was exposed to three conditions to assess the resulting changes in the COP. The COP range of motion while moving forward, to the paralyzed side, to the nonparalyzed side, and backward, and the stable limitation changes were randomly measured while participants were not using any tape, when tape was attached to the tibial muscle, and when tape was attached to the calf muscle to confirm the immediate effects of taping. To exclude any interference from other treatment effects, measurements were performed on days with no treatment plans; 30 min of rest was allowed between measurements.

### 2.8. Measuring Changes in COP

To measure changes in the COP of stroke patients, a decompression platform (AP1153 Biorescue; France) was used. This device was equipped with 1600 pressure detectors in a measurement area of 400 × 400 mm. The participants first stood straight; then, they moved their body according to the direction of an arrow on the screen while standing straight and maintaining balance. The arrow indicated eight directions: front, back, left, right, front left, front right, back left, and back right. When the arrow disappeared from the screen, they returned to the starting position; then, they moved their body in the direction indicated by the second arrow. The researchers ensured that the participants’ feet did not leave the measurement equipment, although the amount of pressure between the left and right sides changed when they were moving their body. The range of motion while moving forward, left, right, and backward were recorded, and the stability limitation, which is the overall range of motion range, was obtained. Because the participants were stroke patients, the range of motion toward the left and right were considered range of motion toward the paretic side and range of motion toward the nonparalyzed side, depending on the patient.

### 2.9. Statistical Analysis

The SPSS 20.0 program (IBM, Armonk, NY, USA) was used for all statistical analyses in this study. All data were analyzed using frequency analysis and descriptive statistics and are expressed as means and standard deviations. Frequency analysis and descriptive statistics were used to assess general characteristics, and normal distribution was obtained using the Shapiro–Wilk test. One-way repeated analysis of variance (ANOVA) was used for changes during each time period according to the taping conditions. If there was a significant change, then Tukey’s postanalysis was performed to verify the results. Statistical significance (level “a”) was set to 0.05.

## 3. Result

**Figure 3:**
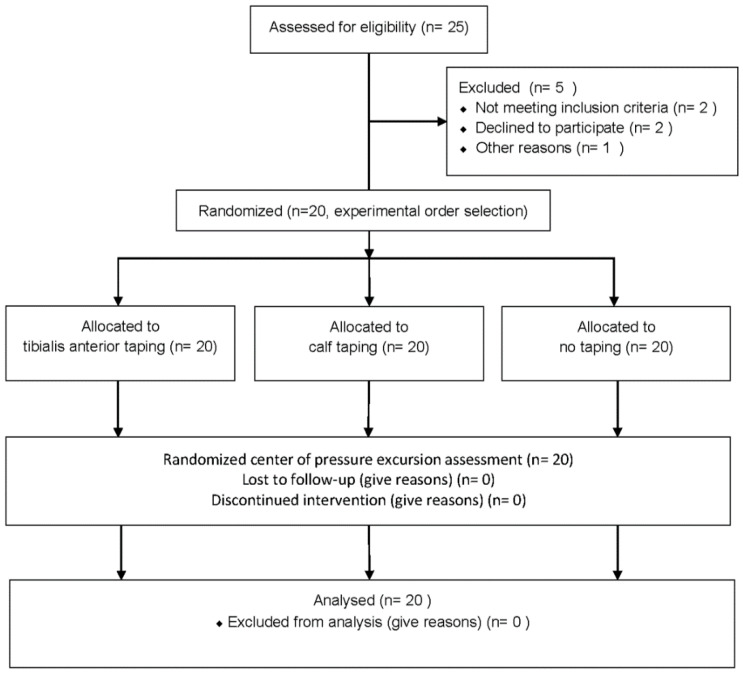
Flowchart of the participant selection.

Twenty-five research participants were selected for this study, but five did not meet the research criteria; therefore, they were excluded. A total of 20 participants were included in this study. The general characteristics of the participants are shown in Table 1 and Figure 3.

**Figure 4:**
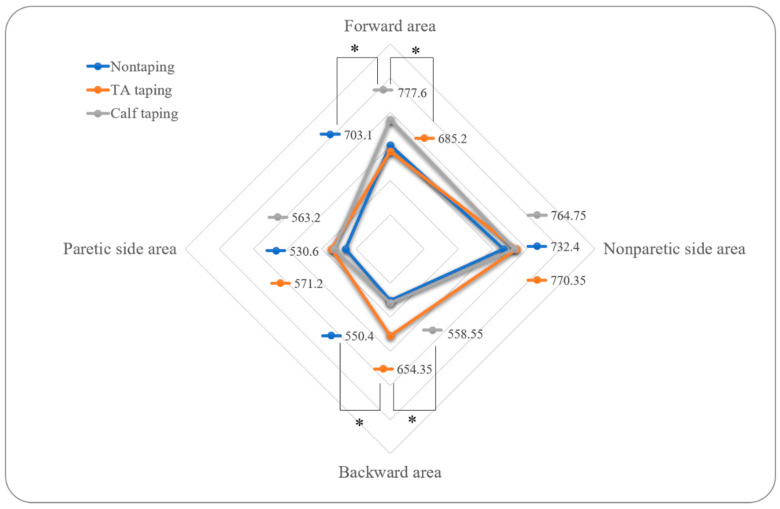
Comparisons of COP excursion for three conditions. TA: tibialis anterior. All values are presented as mean values. * *p* < 0.05.

Figure 4 and Table 2 shows comparisons of each taping condition. When the tape was attached to the calf muscle, there was a significant increase in the forward area compared to the pretaping period and compared to when the tape was attached to the tibial muscle. When the tape was attached to the tibial muscle, there was a significant increase in the backward area compared to the pretaping period and compared to when the tape was on the calf muscle.

## 4. Discussion

This study investigated the use of kinesiology tape [10,12,16] for patients with spasticity of ankle muscles after stroke, to identify the COP change according to the taping attachment method by attaching taping to the tibialis anterior [12] and gastrocnemius [15,16].

There was a significant increase in backward movement when the tape was attached to the tibial muscle, compared to when no tape was used. Furthermore, there was a significant increase in forward movement when the tape was attached to the calf muscle, compared to when no tape was used. Previous study showed that kinesiology tape, which uses 100% elasticity for dorsiflexion and inversion in stroke patients, decreased immediate inversion and eversion COP movements and increased the Berg balance scale score, when compared to the control group without tape [13]. In another study, ankle eversion taping increased velocity, step length, stride length, and cadence, when compared to placebo conditions and conditions without tape [18]. These results are consistent with our findings, which show immediate improvements regarding forward and backward movement, compared to conditions without tape. Thus, kinesiology taping appeared to induce the increasing COP movement.

The dorsiflexion strength through the stretch reflex is important for controlling the backward range of motion, and the plantar flexion strength is important for controlling the forward range of motion [19]. Because dorsiflexion support helped with the backward range of motion, and because plantar flexion support helped with the forward range of motion, our results are similar to those of previous studies. However, previous studies focused on reflexes that occur with quick movements [19], which are different from the voluntary movements that were the focus of this study. Moreover, one study of taping found that when the tape was wrapped around the calf muscle, there was no change in the H–reflex amplitude, which is used to evaluate motoneuron pool activities [20]. However, other studies of taping found that the tape attached to the calf muscle increased the forward range of motion of patients with multiple sclerosis [10], and that when the tape was attached to the back, only torso bending strength increased [21]. Hence, kinesiology tape may have supported movement in the ankles to safely allow patients to move forward and backward.

When an ankle–foot orthosis was used for stroke patients in other studies with an objective similar to that of our study involving taping, the COP sway was reduced during static standing conditions and bilateral limb-loading symmetry increased [22]. Furthermore, the ankle–foot orthoses improved the postural responses of the lateral and backward perturbation directions [23]. In this study, the backward movement improved by taping of the tibial muscle, and forward movement improved by taping of the calf muscle. This improved the patient’s range of motion as it promoted ankle functions by supporting the movements due to the taping [12,13] rather than fixating the ankle joint like applying the brace. Unlike prosthetics, taping does not require high costs or the burden of having to customize and manufacture a new device according to changing body sizes. Taping can be easily used by anyone at an affordable price, which makes it clinically beneficial. Moreover, taping is relatively lightweight, has no issues associated with body weight, and does not limit mobility.

Despite the abovementioned benefits, taping is problematic because lasting effects cannot be expected when the tape is removed [24]. Furthermore, because this study focused on muscle weakness, we were unable to directly verify stiffness, joint range of movement, and muscle activity of stroke patients. Additionally, this study was performed with single group and long-term effects need to be investigated. However, as the forward area of pressure increases, dorsiflexion will be more active, and as the backward area of pressure increases, plantar flexion will become active, thereby indirectly indicating muscle activity [19]. Hence, this study has clinical significance because it verified dynamic balance abilities through the effects of taping.

## 5. Conclusions

In this study, the COP movement of stroke patients was improved by elastic action according to the direction of the attached tape and the attachment point. Specifically, kinesiology tape immediately increased the forward and backward COP excursion for patients with stroke.

## Figures and Tables

**Table 1:** Participant characteristics.

Characteristics	Subjects (n = 20)
Sex (male/female)	14/6
Paretic side (left/right)	9/11
Etiology (infarction/hemorrhage)	13/7
Age (years)	58.95 ± 11.27
Height (cm)	163.30 ± 7.28
Weight (kg)	66.05 ± 6.36
Disease duration (month)	12.35 ± 2.56
K–MMSE (point)	27.60 ± 0.10
K–NIHSS score	9.65 ± 3.25

K–MMSE is the Korean Mini-Mental State Examination; K–NIHSS is the Korean National Institute of Health Stroke Scale.

**Table 2:** Comparisons of center of pressure (COP) excursion for three conditions.

Variable	Before Tape	TA Taping	Calf Taping	F	*p*
Paretic side area	530.60 ± 284.53	571.20 ± 271.40	563.20 ± 272.85	2.065	0.156
Nonparetic side area	732.40 ± 407.92	770.35 ± 443.29	764.75 ± 419.97	1.963	0.154
Forward area	703.10 ± 394.27	685.20 ± 392.86	777.60 ± 386.02 ^a^	5.596	0.013 *
Backward area	550.40 ± 290.83	654.35 ± 328.69 ^b^	558.55 ± 305.57	9.289	0.001 *

TA: tibialis anterior. Values represent mean ± standard deviation. * *p* < 0.05; ^a^ The calf taping conditions were significantly better than pretaping conditions and the tibialis anterior taping conditions. ^b^ The tibialis anterior taping conditions were significantly better than pretaping conditions and calf taping conditions.
